# Seeing is believing! Imaging Ca^2+^-signalling events in living cells

**DOI:** 10.1113/expphysiol.2010.052456

**Published:** 2010-08-09

**Authors:** J Graham McGeown

**Affiliations:** Centre for Vision and Vascular Sciences, Queen's University of Belfast, Institute of Clinical Sciences, Grosvenor Road, Royal Victoria HospitalBelfast BT12 6BA, UK

## Abstract

Ever since it was shown that maintenance of muscle contraction required the presence of extracellular Ca^2+^, evidence has accumulated that Ca^2+^ plays a crucial role in excitation–contraction coupling. This culminated in the use of the photoprotein aequorin to demonstrate that [Ca^2+^]_i_ increased after depolarization but before contraction in barnacle muscle. Green fluorescent protein was extracted from the same jellyfish as aequorin, so this work also has important historical links to the use of fluorescent proteins as markers in living cells. The subsequent development of cell-permeant Ca^2+^ indicators resulted in a dramatic increase in related research, revealing Ca^2+^ to be a ubiquitous cell signal. High-speed, confocal Ca^2+^ imaging has now revealed subcellular detail not previously apparent, with the identification of Ca^2+^ sparks. These act as building blocks for larger transients during excitation–contraction coupling in cardiac muscle, but their function in smooth muscle appears more diverse, with evidence suggesting both ‘excitatory’ and ‘inhibitory’ roles. Sparks can activate Ca^2+^-sensitive Cl^−^ and K^+^ currents, which exert positive and negative feedback, respectively, on global Ca^2+^ signalling, through changes in membrane potential and activation of voltage-operated Ca^2+^ channels. Calcium imaging has also demonstrated that agonists that appear to evoke relatively tonic increases in average [Ca^2+^]_i_ at the whole tissue level often stimulate much higher frequency phasic Ca^2+^ oscillations at the cellular level. These findings may require re-evaluation of some of our models of Ca^2+^ signalling to account for newly revealed cellular and subcellular detail. Future research in the field is likely to make increasing use of genetically coded Ca^2+^ indicators expressed in an organelle- or tissue-specific manner.

## Live cell imaging: a terrible beauty

**Figure 1:**
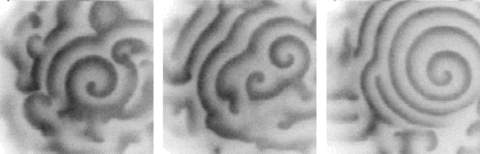
Calcium ion spiral waves in *Xenopus* oocytes Three frames have been chosen from a movie of an oocyte to show how the wave patterns evolve over time. These spectacularly patterned signals were first described by Lechleiter *et al.* (1991). Reproduced with permission of Professor James Lechleiter PhD: http://www.uthscsa.edu/csb/faculty/lechleiter.asp.

Imaging biological processes in living cells has had and will continue to have great impact on our scientific understanding of biological function. I believe it also has an amazing and largely untapped potential to stir the imagination of society at large by presenting the world of cell biology in an accessible and aesthetically stimulating way. Just as the amazing images from the Hubble telescope make cosmologists of us all, so too advances in imaging technology have provided us with pictures dramatic enough to engage and enthuse even those with the most limited interest in biology. The invisible has been made visible, revealing unsuspected beauty and endless movement. This is dramatically exemplified by the Ca^2+^ waves seen in *Xenopus* oocytes expressing muscarinic acetylcholine receptors (Fig. 1; Lechleiter *et al.* 1991). These spiral across the cell in endlessly evolving patterns, never repeating the same sequence twice but always obeying simple rules of propagation and annihilation. I will let this example stand for now not just as a great piece of science but also as a thing of wonder in its own right, easily appreciated by anyone who has ever stood delighted by a Catherine-wheel's fiery corkscrewing against the night sky.

## What is so special about Ca^2+^ anyway?

**Figure 2:**
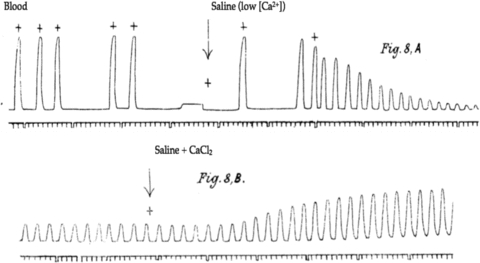
An early demonstration of the importance of Ca^2+^ for muscle contractility Changes in volume were recorded from an isolated frog ventricle perfused with blood or saline solutions (more detail on the technique and Ringer's work is presented by Miller, 2004). Full contractions were only seen when Ca^2+^ was present in the perfusate. Figure reproduced with permission from Ringer (1883).

Before looking at Ca^2+^ imaging itself, I want to provide a very brief and ridiculously selective overview of the evidence pointing to the importance of Ca^2+^ signalling. Although free Ca^2+^ inside the cytosol is a very small percentage of total body Ca^2+^, most of which is found in bone mineral, it is a crucial cell signal in many cell types. Some of the earliest evidence for this came from experiments carried out in the 1880s on frog heart muscle by Sydney Ringer. He wanted to develop a salt solution suitable for *in vitro* studies on isolated tissues. Early experiments were promising, but subsequent attempts to repeat the work gave much less satisfactory results. Some detective work led to the discovery (Ringer, 1883) ‘that the saline solution which I had used’ (i.e. in the earlier successful study) ‘had not been prepared with distilled water, but with pipe water supplied by the New River Water Company … . . It is obvious therefore that the effects I had obtained are due to some of the inorganic constituents of the pipe water.’ It is generally accepted that Ringer's technician was probably responsible for the initial oversight in using pipe water rather than distilled water, but the consequences were positive, since they demonstrated both that the heart could be kept beating and that this required some ingredient missing from saline prepared with distilled water (Miller, 2004). Ringer went on to determine which of the ‘inorganic constituents’ in pipe water was important and discovered that his artificial saline solution only supported consistent cardiac contractions when Ca^2+^ was present, suggesting that this was necessary for normal cell function (Fig. 2). By such serendipity knowledge is advanced.

**Figure 3:**
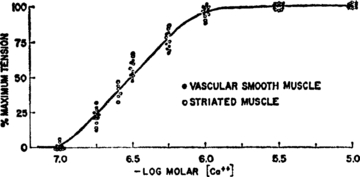
Effect of changing [Ca^2+^] on contraction in permeabilized muscle Permeabilized muscle fibre preparations were used, allowing intracellular [Ca^2+^] to be controlled simply by changing the extracellular [Ca^2+^]. The otherwise intact fibres developed active tension when [Ca^2+^] exceeded 100 nm, with maximal contraction above 1 μm. Interestingly, the relationships in striated and smooth muscles were almost identical. Reproduced with permission from Filo *et al.* (1965).

Since these early observations, a large amount of evidence has accumulated suggesting that changes in intracellular [Ca^2+^] ([Ca^2+^]_i_) play a crucial role in the control of cell function, particularly in muscle. The sliding filament hypothesis of striated muscle contraction was established independently (by two different Huxleys) based on structural and functional experiments in the 1950s (see review, Huxley, 2000). By the early 1960s it was apparent that biochemical extracts of the contractile machinery could interact in the test tube to form a superprecipitate. This process required millimolar MgATP as an energy source but was also highly sensitive to [Ca^2+^], with measurable superprecipitation and ATP breakdown when [Ca^2+^] exceeded 10^−7^m (Weber & Winicur, 1961). This mimicking of muscle contraction in a test tube suggested that Ca^2+^ played some sort of role in controlling the contractile process. The development of techniques allowing muscle cells to be permeabilized, so that intracellular and extracellular [Ca^2+^] become equal, demonstrated a very similar relationship between [Ca^2+^]_i_ and contractile force in permeabilized fibres and the Ca^2+^ dependence of superprecipitation, with activation of muscle contraction when [Ca^2+^]_i_ exceeds 100 nm and maximal contraction above 1 μm (Fig. 3; Filo *et al.* 1965). Interestingly, the same relationship applied to both smooth and striated muscle, even though we now know there are major differences in the way contraction is regulated in these different muscle types (Fig. 3). We will return to this point later.

The evidence up to this point supported a model in which excitation of a striated muscle fibre by the propagation of an action potential across the cell membrane was coupled to mechanical contraction via the intracellular machinery through a Ca^2+^-dependent signalling process. However, this evidence was largely indirect and so the hunt was now on for some way to record changes in [Ca^2+^]_i_ during electrically evoked muscle contraction in intact cells, so as to more closely reflect physiological events.

## Measurements of [Ca^2+^]_i_ in living cells

**Figure 4:**
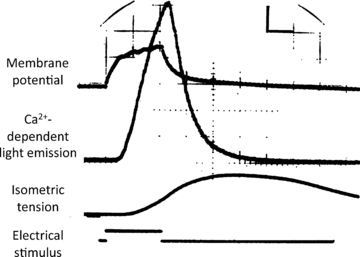
A direct demonstration that Ca^2+^ is involved in excitation–contraction coupling Barnacle muscle fibres were injected with aequorin, a protein which chemiluminesces when it binds Ca^2+^. The fibres were stimulated electrically, mimicking depolarization by an action potential, and changes in membrane potential, [Ca^2+^] (light generated by aequorin) and tension were measured simultaneously. Depolarization, a rise in [Ca^2+^]_i_ and contraction occurred in that sequence, consistent with the hypothesis that Ca^2+^ signalling plays an important role in excitation–contraction coupling. Reproduced with permission from Ashley & Ridgway (1970).

The relevant information was eventually derived from experiments using a Ca^2+^ indicator from a most unlikely source. Certain species of luminescent jellyfish manufacture the photoprotein aequorin, which emits a blue glow when it interacts with Ca^2+^. The protein was first isolated and purified from *Aequoria aequoria* by Osamu Shimomura, a biochemist with a keen interest in light-emitting molecules from biological sources (bioluminescence), while working in the laboratory of Frank Johnson at Princeton. They showed that the amount of light released could be used as a measure of the [Ca^2+^] in an aequorin-containing solution, opening the way for biological applications of the technique (Shimomura *et al.* 1963). Aequorin was used to demonstrate that Ca^2+^ signalling played a key role in excitation–contraction coupling within barnacle muscle (Ashley & Ridgway, 1968, 1970). This has large-diameter fibres, greatly facilitating intracellular aequorin injection. Contractions were stimulated by electrically depolarizing the fibres, and the resulting changes in membrane potential, Ca^2+^-dependent light emission by the aequorin and contractile force measured simultaneously (Fig. 4). This allowed both the amplitudes and the temporal sequencing of these events to be assessed. Membrane depolarization was followed by a rise in [Ca^2+^], which was, in turn, followed by muscle contraction. Since cause must precede effect, this ruled out the possibility that the [Ca^2+^]_i_ increase was some sort of epiphenomenon or ‘side-effect’ of contraction itself, but was consistent with the hypothesis that excitation of the muscle fibre causes an increase in intracellular [Ca^2+^], which activates the formation of cross-bridges between myosin and actin. We now know that, in striated muscle, this results from the binding of Ca^2+^ to the troponin–tropomyosin complex on the thick myofilament, thereby relieving steric inhibition of cross-bridge formation between myosin heads and actin (Galinska-Rakoczy *et al.* 2008). Calcium also plays an important role in smooth muscle signalling using very different downstream mechanisms, with Ca^2+^–calmodulin activating myosin light chain kinase. The resulting phosphorylation upregulates myosin ATPase activity, releasing the chemical energy needed for cross-bridge cycling and cell shortening (Itoh *et al.* 1989).

This work established the involvement of intracellular Ca^2+^ in excitation–contraction coupling. Aequorin was difficult to use, however, since it had to be isolated and purified from jellyfish and then injected into each cell to be studied. The only alternative approach, which involved impaling cells with a Ca^2+^-selective microelectrode, was equally laborious and technically difficult, and could not always follow very rapid changes in [Ca^2+^]_i_ (Lee, 1981). Better, more user-friendly Ca^2+^ indicators were clearly needed. This challenge was identified and taken on by Roger Tsien, first in the University of Cambridge and later in the University of California, Berkeley. He recognized that, just as physiologists had been obliged to design and manufacture the electronic equipment needed for the study of neuronal action potentials in a previous generation, so too there was a need to develop the new chemical tools needed for cell-signalling research. A range of fluorescent, Ca^2+^-sensitive dyes resulted from this work, along with a chemical strategy allowing them to be easily introduced into intact cells (Kresge *et al.* 2006). Acetoxymethyl esters are lipid soluble and cell permeant but are readily hydrolysed by intracellular enzymes, releasing the active dye in its anionic form. This remains trapped within the cell, and changes in fluorescence can then be used as a measure of [Ca^2+^]_i_. Based on its use in research papers, the most successful of these compounds was fura-2, a ratiometric Ca^2+^ indicator. These dyes circumvent movement artefacts and open the door to calibration of the signal in terms of absolute [Ca^2+^] (Grynkiewicz *et al.* 1985).

As is often the case, the availability of new research tools that were relatively easily applied led to a massive explosion in Ca^2+^-signalling research, with many thousands of papers published in the last 25 years. It quickly became apparent that increases in [Ca^2+^]_i_ were involved in the transduction of external stimuli into cellular responses in almost every cell type studied. Calcium signalling is genuinely ubiquitous, affecting a wide variety of molecular targets in many different types of cells. No one reference can really capture this diversity, but a simple PubMed search for the term ‘Ca-signalling’ generated over 7900 hits in June 2010. The importance of Ca^2+^ signalling is indicated by the very low basal [Ca^2+^]_I_ found in most cells, which is maintained by a variety of Ca^2+^ pumps and exchangers. This allows relatively small concentration increases to be resolved by Ca^2+^-sensitive proteins. An analogy can be found in astronomy, in which viewing conditions improve as background ‘light pollution’ decreases, making faint objects easier to detect. At about 100 nm or so, resting [Ca^2+^]_i_ is more than four orders of magnitude lower than the free extracellular [Ca^2+^]_i_ in mammals, and at least 100 times less than that in the sarcoplasmic/endoplasmic reticulum, the major intracellular Ca^2+^ store (McCarron *et al.* 1992; Wray & Burdyga, 2010). Despite the large electrochemical gradients involved, however, Ca^2+^ does not simply flood into the cytosol, since it cannot readily cross lipid membranes. Calcium can only diffuse into the cell if appropriate ion channels are activated, providing a Ca^2+^-permeable access route. Many such channels have been identified in the cell membranes of both excitable and non-excitable tissues, and are activated by very diverse stimuli. Intracellular stores can release Ca^2+^, mainly via opening of ryanodine receptor (RyR)-gated or inositol 1,4,5-trisphosphate receptor (IP_3_R)-gated release channels. Some of these Ca^2+^ channels will be mentioned in what follows, but no attempt will be made to provide an overview of this vast field.

## Bioluminescence and biofluorescence: a Nobel calling

Before going on to consider the new insights gained from Ca^2+^ imaging itself, it is worth mentioning the close conceptual and historical connections between the development of Ca^2+^ indicators and other live cell imaging techniques based on fluorescent protein markers. In Shimomura and Johnson's original work on *Aequoria*, they noticed that the jellyfish produced a second optically active protein, which generated green fluorescence when excited by the light emitted from the aequorin itself (Shimomura, 2009). At the time, this work was simply motivated by the researchers’ interest in the mechanisms responsible for light production in animals, and the discovery of green fluorescent protein (GFP) in 1962 (Shimomura *et al.* 1962) remained of apparently limited interest for over 30 years. However, the cloning of the GFP gene in 1992 led to its expression in the nematode in 1994, the first demonstration that it could be used as a marker of gene expression in living cells (Prasher *et al.* 1992; Chalfie *et al.* 1994). By introducing the DNA code for GFP linked to a gene of choice, the expression and trafficking of any specified protein could now be visualized in living cells. Roger Tsien applied his understanding of fluorescent chemistry, first stimulated by the need to design improved Ca^2+^ indicators, to develop a range of modified fluorescent proteins, allowing different gene products to be imaged simultaneously in the same organism or cell (Tsien, 2010). This work led to the rapid and diverse application of live cell imaging to address key questions in biology, a technical revolution for which Shimomura, Chalfie and Tsien were awarded the 2008 Nobel Prize for Chemistry (Chalfie, 2009; Shimomura, 2009; Zimmer, 2009; Tsien, 2010). Once again, new tools led to new knowledge. All of this came from Shimomura and Johnson's fascination with creatures that glow. At the time, neither they nor anyone else could have guessed what amazing scientific and technological implications their findings would have. They just wanted to know! Perhaps this stands as a reminder that we need to leave room for curiosity-driven enquiry, no matter how little obvious application the work might have, since nobody can foretell where the next crucial finding will come from. If we could, it wouldn't really be research.

## Confocal technology, Ca^2+^ imaging and Ca^2+^ sparks

**Figure 5:**
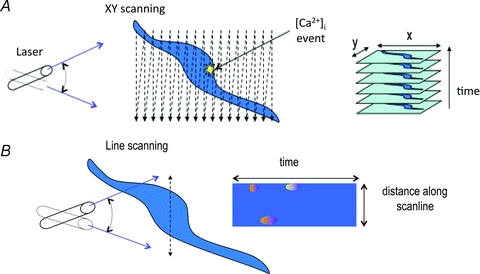
Application of confocal microscopy to Ca^2+^ imaging in living cells Cells are loaded with a fluorescent Ca^2+^ indicator (e.g. fluo-4) and excited with a scanning laser. *A*, in *XY* scanning mode a single image plane is scanned repeatedly, allowing any [Ca^2+^]_i_ rises to be recorded and their location identified. *B*, line scanning greatly increases the speed of acquisition by imaging only along a single line rather than a full *XY* plane. The resulting images show how [Ca^2+^]_i_ changes locally at each point on the line as time passes. This is particularly useful when imaging fast events, such as Ca^2+^ sparks.

Getting back to the Ca^2+^ story, the step from Ca^2+^ measurement to Ca^2+^ imaging came with the development of new microscopy techniques. Recordings using Ca^2+^ indicators often involve a technique referred to as microfluorimetry, in which the overall (i.e. spatially summed) intensity of any emitted fluorescence is recorded. Although a microscope objective is used to collect the light, no fluorescent image is captured. There are several technical reasons for doing this, including increased sensitivity and speed of recording, important with rapidly changing signals. However, the development of confocal microscopy and ever more sensitive CCD-based cameras made true Ca^2+^ imaging a viable option, in which local changes in [Ca^2+^]_i_ within individual cells can be visualized. Confocal microscopes combine a scanning laser to excite cell fluorescence with a pinhole to exclude out-of-focus light coming back into the imaging scanhead, allowing clear optical sections to be recorded from much thicker tissues. Initially, this technique was used to improve the resolution of images from fixed specimens stained with fluorescent dyes, and to generate three-dimensional tissue models by imaging different planes in the same sample and combining the data (Brakenhoff *et al.* 1989). It quickly became obvious, however, that this technology was also ideal for Ca^2+^ imaging using fluorescent indicators in living cells (Niggli & Lederer, 1990). Rather than simply recording the average signal emitted from the sample, as is the case in Ca^2+^ microfluorimetry, it was now possible to repeatedly image the Ca^2+^-dependent fluorescence in a given optical section of a cell or tissue, generating a ‘movie’ showing how Ca^2+^ changed over time in different cells and even in different parts of the same cell (Fig. 5). The only problem with these recordings was that they were too slow to faithfully reproduce the rapid changes in [Ca^2+^]_i_ seen in muscle cells, which were the first focus of interest. This limitation could be largely overcome by adapting the technique so that the laser did not scan across the whole of the image plane but repeatedly scanned back and forth along a single line. Temporal resolution was greatly increased (from about 1 frame s^−1^ to about 500 lines s^−1^), but at the cost of one spatial dimension. This sacrifice was soon shown to be justified, however, as the resulting linescan images revealed subcellular Ca^2+^ events previously unsuspected in cardiac muscle cells. These brief, spatially localized increases in [Ca^2+^]_i_ were dubbed ‘Ca^2+^ sparks’ and were shown to represent spontaneous release of Ca^2+^ from the sarcoplasmic reticulum via cardiac ryanodine receptors (Cheng *et al.* 1993). It should also be noted in passing that localized spontaneous Ca^2+^-release events mediated by clusters of IP_3_Rs have also been identified in a range of excitable and non-excitable tissues and given the name ‘Ca^2+^ puffs’ (Smith *et al.* 2009; Wray & Burdyga, 2010). These will not be considered further.

**Figure 6:**
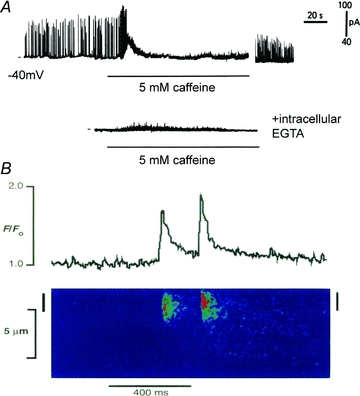
Indirect and direct evidence for Ca^2+^ sparks in smooth muscle *A*, electrophysiological recordings from voltage-clamped smooth muscle reveals spontaneous outward transient currents (STOCs) carried by K^+^. Activation of RyRs with caffeine to release the sarcoplasmic reticulum Ca^2+^ store resulted in an initial burst of STOC activity and then quiescence. Inclusion of EGTA in the recording pipette to buffer changes in [Ca^2+^]_i_ inhibited STOCs. This indirect but highly persuasive evidence suggests that there must be spontaneous Ca^2+^ release via RyRs, resulting in the activation of Ca^2+^-activated BK channels. Reproduced with permission from Benham & Bolton (1986). *B*, confocal linescan image of an arterial smooth muscle cell loaded with the indicator fluo-3. Normalized fluorescence (*F*/*F*_0_) is used as a measure of [Ca^2+^]_i_. Two spontaneous Ca^2+^ sparks have been recorded. Reproduced with permission from Nelson *et al.* (1995).

We will return to the function of Ca^2+^ sparks later, but first I want to consider smooth muscle, which will be the main focus for the rest of this discussion. Interestingly, evidence that something similar to Ca^2+^ sparks must occur in smooth muscle had been reported nearly a decade before they were imaged. Electrophysiological recordings, in which the membrane potential of a smooth muscle cell could be clamped at a specific voltage and transmembrane ion currents recorded, had revealed brief, relatively short-lived and outwardly directly K^+^ currents (Fig. 6*A*). These were given the descriptive title ‘spontaneous transient outward currents (STOCs)’ (Benham & Bolton, 1986). When caffeine was applied to release stored Ca^2+^ by activating RyRs, there was an initial burst of STOC activity, followed by relative quiescence. This response could be inhibited by introducing EGTA, a Ca^2+^ chelator, to the intracellular solution via the recording pipette, indicating that the current was activated by a rise in [Ca^2+^]_i_. Taken together, these findings suggested that STOCs reflected spontaneous Ca^2+^ release from the sarcoplasmic reticulum via RyRs, leading to the activation of Ca^2+^-sensitive K^+^ channels conducting the outward current (*I*_KCa_). Such channels (also known as large-conductance K^+^ channels, or BK channels) had already been identified in smooth muscle (Walsh & Singer, 1981; Benham *et al.* 1986). It was not until 1995, however, that this indirect evidence for localized spontaneous Ca^2+^ release in smooth muscle was confirmed by the direct visualization of Ca^2+^ sparks, again using confocal imaging of Ca^2+^ indicator fluorescence (Fig. 6*B*; Nelson *et al.* 1995). Both sparks and STOCs were inhibited by using ryanodine to block RyR activity and by agents that depleted the Ca^2+^ stores. Calcium imaging therefore allowed spontaneous Ca^2+^ release to be directly observed in smooth muscle cells, confirming a hypothesis originally postulated on the basis of indirect evidence.

## What do sparks do?

Physiology is driven by functional questions. Usually, this expresses itself in the desire to understand the mechanisms responsible for the function of a molecule, cell, organ or organism. In the case of Ca^2+^ sparks, however, we are presented with a cell mechanism whose function needs to be defined. In the case of cardiac muscle, it was immediately proposed that they might act as fundamental building blocks from which larger and global Ca^2+^ transients are constructed during the excitation–contraction process (Cheng *et al.* 1993). This view has been confirmed over the intervening years. Depolarization of cardiac myocytes activates voltage-operated (L-type) Ca^2+^ channels in the plasma membrane, leading to an influx down the electrochemical gradient from the extracellular fluid. This stimulates the opening of near-membrane RyRs, which are directly sensitive to the local [Ca^2+^]_i_, leading to an almost synchronous cell-wide Ca^2+^ rise and contraction (Cheng & Lederer, 2008). Individual sparks are not easily identified in confocal linescans of normal Ca^2+^ transients but become obvious when relatively small depolarizations are applied. Thus, in cardiac muscle at least, sparks act as excitatory events whose summation generates the intracellular signal responsible for contraction.

**Figure 7:**
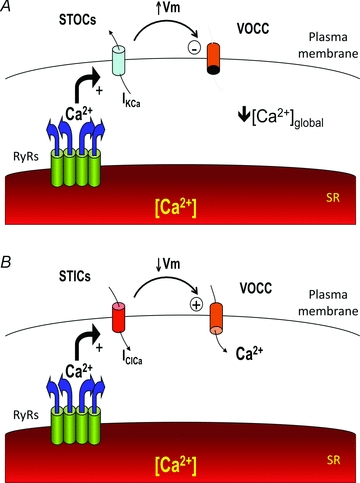
Sparks activate Ca^2+^-sensitive membrane currents in smooth muscle *A*, activation of Ca^2+^-activated K^+^ channels by Ca^2+^ sparks results in spontaneous transient outward currents (STOCs), hyperpolarizing the plasma membrane (increased membrane potential; *V*_m_). This reduces activation of voltage-operated Ca^2+^ channels (VOCCs), decreasing Ca^2+^ influx and global [Ca^2+^]. This can act as a negative feedback mechanism, whereby localized Ca^2+^ release from the sarcoplasmic reticulum (SR) lowers mean cytosolic [Ca^2+^], restricting further store loading. *B*, spark-dependent activation of Ca^2+^-activated Cl^−^ channels causes spontaneous transient inward currents (STICs), with membrane depolarization and Ca^2+^ channel activation. This results in positive feedback, with Ca^2+^ release from the SR promoting further Ca^2+^ influx.

The story in smooth muscle, however, does not appear to be so straightforward. In the initial report of Ca^2+^ sparks in arterial smooth muscle, considerable evidence was provided for an inhibitory function. Interventions that inhibited sparks resulted in constriction of pressurized arteries, suggesting that sparks normally exert a dilatory influence (Nelson *et al.* 1995). It was proposed that this reflects negative feedback via spark activation of BK channels, resulting in outward, hyperpolarizing current (Fig. 7). Activation of voltage-operated Ca^2+^ channels is reduced in these circumstances, limiting Ca^2+^ influx. Localized Ca^2+^ release in the form of Ca^2+^ sparks could, therefore, favour a reduction in global [Ca^2+^]_i_, relaxing smooth muscle. Considerable evidence supports this model in many different smooth muscles, particularly in vascular and urinary myocytes (Perez *et al.* 2001; Heppner *et al.* 2003; Burdyga & Wray, 2005; McGahon *et al.* 2007).

**Figure 8:**
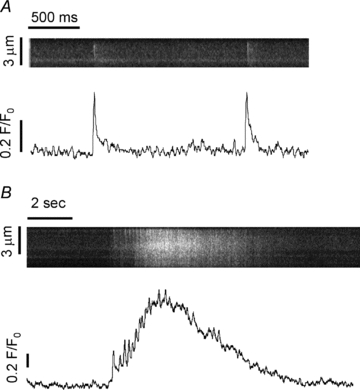
Calcium sparks and oscillations recorded from retinal arteriole myocytes *in situ* Arterioles were loaded with the Ca^2+^ indicator fluo-4, and scanned transversely across their diameter along a single scanline 500 s^−1^ using a confocal laser microscope (see Fig. 9*B*). Normalized fluorescence (*F*/*F*_0_) is a measure of local [Ca^2+^]. *A*, two spontaneous Ca^2+^ sparks, with a rapid rising phase and slower decay. The entire event typically lasts <150 ms. *B*, a more prolonged Ca^2+^ oscillation lasting several seconds. These are sometimes associated with myocyte contraction. Spark activity is clearly seen near the upper cell margin, and sparks appear to summate during the rising phase of the oscillation. For more detail see Curtis *et al.* (2004) and Tumelty *et al.* (2007). Image courtesy of Dr Tim Curtis.

This is conceptually quite different from their proposed role in cardiac muscle, where sparks are used to construct global Ca^2+^ signals. Evidence is now accumulating, however, to suggest that Ca^2+^ sparks may also play an excitatory role in some smooth muscles. We have used confocal linescan imaging to investigate Ca^2+^ signalling in the smooth muscle of retinal arterioles, which regulate blood flow to the inner retina. These myocytes generate both brief localized Ca^2+^ sparks and more prolonged Ca^2+^ oscillations (Fig. 8). Rather than reducing global [Ca^2+^]_i_, sparks often appear to summate, generating oscillations, which sometimes result in obvious contraction of the relevant myocyte (Curtis *et al.* 2004). In more recent experiments using faster two-dimensional Ca^2+^ imaging, Ca^2+^ sparks again appear to initiate cell-wide Ca^2+^ oscillations and contraction, again suggesting an excitatory rather than an inhibitory role (Tumelty *et al.* 2007). Pharmacological interventions that reduce spark frequency also reduce spontaneous oscillations, and constrictor agonists increase both sparks and oscillations (Tumelty *et al.* 2007; Jeffries *et al.* 2010). Similar observations have been made by other groups, with intracellular sites that demonstrate high levels of spontaneous spark activity (termed ‘frequent discharge sites’) acting as points of initiation for cell-wide spontaneous Ca^2+^ waves and evoked Ca^2+^ transients. This suggests that, as in cardiac muscle, sparks in smooth muscle can also act as ‘elementary building blocks’, from which excitatory global Ca^2+^ signals are constructed (Bolton *et al.* 2004). It should also be noted that, although sparks can exert negative feedback by activating outward *I*_KCa_, the presence of Ca^2+^-activated Cl^–^ channels in the plasma membrane also opens up the possibility of positive feedback following a rise in [Ca^2+^]_i_ (Fig. 7; Large & Wang, 1996; Jackson, 2000; McGahon *et al.* 2009). In smooth muscle, Cl^−^ currents are inwardly directed at normal resting potentials (outward movement of negatively charged Cl^−^ ions equates to inward conventional current). The resulting spontaneous transient inward currents (STICs) tend to depolarize the plasma membrane, increasing the steady-state activation of voltage-operated Ca^2+^ channels and so raising cell [Ca^2+^]_i_. Calcium sparks have been shown to generate STICs in a number of different smooth muscle types (Bao *et al.* 2008). Indeed, depending on the conditions, a single spark can activate a STOC followed by a STIC. The resulting biphasic current has been rather philosophically dubbed a STOIC (ZhuGe *et al.* 1998). The functional consequences of such complex signals are only just beginning to be explored (ZhuGe *et al.* 2010).

## What you see depends on how you look at things: agonist stimulation and Ca^2+^ oscillations

**Figure 9:**
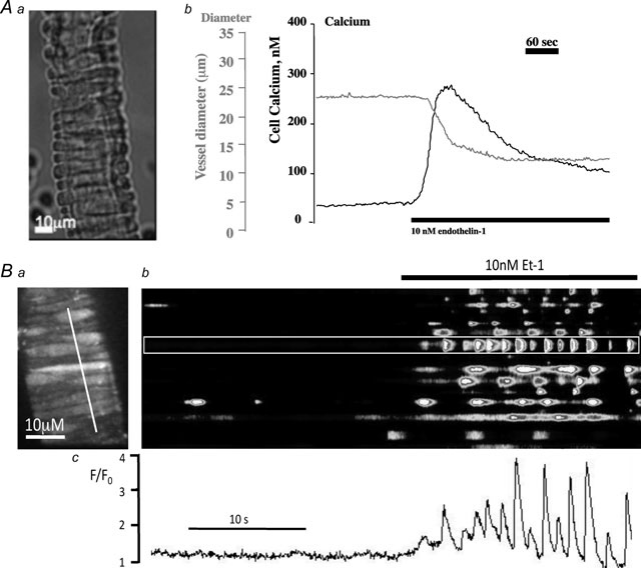
Calcium imaging reveals cell behaviour not apparent in ‘average’ recordings *A*, a retinal arteriole (*Aa*) was loaded with fura-2 AM and changes in mean [Ca^2+^]_i_ and vessel diameter recorded (*Ab*). This technique records changes in smooth muscle but not endothelial [Ca^2+^] (Scholfield & Curtis, 2000). Addition of endothelin-1 (Et-1; 10 nm) leads to an increase in [Ca^2+^]_i_, followed by constriction. There is an initial transient Ca^2+^ peak, followed by a sustained rise but at a lower [Ca^2+^]_i_. Images courtesy of Drs C. N. Scholfield and T. M. Curtis. *B*, confocal Ca^2+^ imaging of retinal arteriole myocytes *in situ* within the vessel wall (*Ba*). Fluo-4-loaded cells have been imaged in a plane near the bottom of the organ bath, revealing an array of adjacent myocytes. These were then scanned along the indicated line to generate a linescan (*Bb*). Addition of endothelin stimulates asynchronous Ca^2+^ oscillations in adjacent cells. The temporal pattern of the cell response (*Bc*) does not mirror that of the global signal seen in *A* (note the faster time base in *B*).

**Figure 10:**
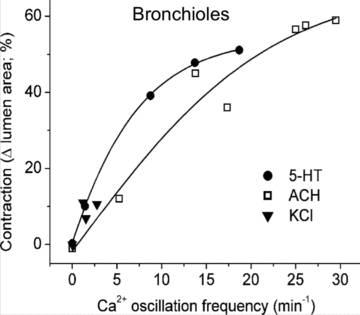
Contraction of bronchiolar smooth muscle is a function of Ca^2+^ oscillation frequency The results were obtained by using Ca^2+^ imaging in pulmonary slices and applying different concentrations of 5-hydroxytryptamine (5-HT) or acetylcholine (ACH), or by using K^+^ to depolarize the smooth muscle (KCl). Reproduced with permission from Perez & Sanderson (2005*a*). Qualitatively similar results were obtained for pulmonary arterioles (Perez & Sanderson, 2005*b*).

Another aspect of Ca^2+^ signalling in smooth muscle of considerable interest relates to its role in control of contraction by neurotransmitters and endocrine or paracrine messenger molecules. We have been interested in how endothelin-1 (Et-1), a highly potent endothelium-derived vasoconstrictor, regulates Ca^2+^ signals in retinal arterioles. Recording the average smooth muscle [Ca^2+^]_i_ using microfluorimetry results in a relatively simple response, with an initial Ca^2+^ transient lasting approximately 100 s, which then falls back towards the control level but remains elevated above baseline (Fig. 9*A*). This triggers a sustained vessel constriction (Curtis *et al.* 2007). The intuitive model of stimulus–contraction coupling suggested by this result is one in which muscle contraction tracks the increase in [Ca^2+^]_i_, during the rising phase at least, consistent with the data from skinned muscle showing that tension is a function of the steady-state [Ca^2+^]_i_ (Fig. 3). Calcium imaging reveals a very different picture, however, in which endothelin-1 stimulates phasic Ca^2+^ oscillations with a much shorter period (<5 s) than the transient seen at the whole vessel level (Fig. 9*B*; Curtis *et al.* 2007). Presumably, the asynchronous nature of these oscillations accounts for the much slower and relatively tonic increase in [Ca^2+^]_i_ recorded using microfluorimetry. Importantly, no individual cell experiences the temporal profile of [Ca^2+^]_i_ changes recorded in the spatially averaged recording. Any realistic model of cell signalling in response to Et-1 must, therefore, be able to account for the more complex pattern revealed by high-speed imaging. Similar oscillatory responses to constricting stimuli, first reported in vascular tissue in 1994 (Iino *et al.* 1994), have subsequently been described in a variety of other smooth muscles. Studies on pulmonary arteriolar and bronchiolar smooth muscle, for example, have demonstrated that increasing concentrations of agonist lead to an increase in the frequency of Ca^2+^ oscillations, and that the resulting contractions can be plotted as a function of frequency rather than ‘mean’[Ca^2+^]_i_ (Fig. 10; Perez & Sanderson, 2005*a*,*b*;). Although high agonist concentrations lead to persistent elevation of global [Ca^2+^]_i_ in most cells, it is reasonable to argue that Ca^2+^ imaging has revealed a major ‘digital’, ‘frequency-modulated’ aspect to smooth muscle Ca^2+^ signalling previously unsuspected from global measurements of average [Ca^2+^]_i_ (Sanderson *et al.* 2010). Calcium ion spikes and oscillations had been demonstrated in a range of cell types, both excitable and non-excitable, prior to the widespread use of Ca^2+^ imaging (Thorn *et al.* 1993; Friel, 1995), but the ability to observe Ca^2+^ changes within individual myocytes embedded in their parent tissue using confocal techniques has demonstrated their importance in smooth muscle in a way not previously appreciated from Ca^2+^ microfluorimetry records. It remains to be seen what functional benefit results from the use of complexly patterned Ca^2+^ signals rather than simple variations in steady-state [Ca^2+^]_i_, although oscillations may allow for intermittent activation of Ca^2+^-dependent processes with a relatively high Ca^2+^ threshold while reducing the risk of cell damage (Berridge *et al.* 2000). The temporal patterning of Ca^2+^ signals may also contribute to signal targeting within the cell just as much as their spatial distribution. This may even play a role in the regulation of protein expression in smooth muscle by Ca^2+^ signals, a process referred to as ‘excitation–transcription coupling’ (Wamhoff *et al.* 2006).

## Conclusion

The application of Ca^2+^-imaging technology has radically altered the way we have to think about signalling at the cellular level. The complex and highly dynamic responses evoked in smooth muscle by agonists, for example, were not obvious in microfluorimetry recordings of average [Ca^2+^], and suggest that frequency modulation of phasic Ca^2+^ waves and oscillations plays an important role in determining the contractile response. Subcellular detail has also been revealed, with brief, spatially localized Ca^2+^-release events. These Ca^2+^ sparks can contribute directly to the elevation of cell [Ca^2+^] and contraction, as they do in cardiac excitation–contraction coupling and in some smooth muscles, or indirectly through activation of Ca^2+^-sensitive membrane conductances, altering membrane potential and thus regulating Ca^2+^ influx via changes in activation of voltage-operated channels. As is usual in science, the ability to look at Ca^2+^ signals in more detail has generated as many new questions as answers, and the challenge of integrating the relevant detail into functional models at the cell and tissue level will be considerable. Future developments will probably include increased use of genetically coded Ca^2+^ indicators, in which proteins with Ca^2+^-sensitive fluorescent or luminescent properties are expressed in specific organelles or tissues (Alvarez & Montero, 2002; Kotlikoff, 2007). This reflects the same basic strategy that underpinned the initial use of aequorin, but with molecules genetically engineered to increase the range of possible applications and target indicator expression to specific cell types or organelles (Eglen & Reisine, 2008).
